# The gut microbiome as a major source of drug-resistant infections: emerging strategies to decolonize and target the gut reservoir

**DOI:** 10.3389/fcimb.2025.1692582

**Published:** 2025-11-05

**Authors:** Ishrya Sharma, Drishya Sudarsanan, Shannon Moonah

**Affiliations:** ^1^ Department of Medicine, University of Florida, Gainesville, FL, United States; ^2^ Department of Molecular Genetics and Microbiology, University of Florida, Gainesville, FL, United States

**Keywords:** antibiotic resistance, gut microbiome, bacteria, fecal micriobiota transplantation, bacteriophage, antimicrobial pepides, probiotics, dietary interventions

## Abstract

Infections caused by antimicrobial-resistant bacteria represent a significant global health crisis that continues to worsen, creating an urgent need for alternative treatment and prevention strategies. A major source of drug-resistant bacteria is the human gut. The gut microbiota consists of bacteria that are frequently exposed to antibiotics, leading to selective pressure that promotes the development of resistant strains such as carbapenem-resistant Enterobacterales (CRE) and vancomycin-resistant enterococci (VRE). These drug-resistant bacteria can spread from the gut to other body sites, leading to hard-to-treat and potentially life-threatening infections such as bacteremia, surgical site infections, and urinary tract infections. Targeting the gut reservoir is essential in the fight against antimicrobial resistance. In this review, we focus on emerging non-antibiotic strategies aimed at eliminating drug resistant bacteria from the gut before they cause invasive infections, with particular emphasis on clinical evidence. Approaches discussed include fecal microbiota transplantation, bacteriophage therapy, antimicrobial peptides, probiotics, and dietary interventions. Optimizing these strategies, while continuing to explore newer approaches, will be essential to combat the growing threat of drug-resistant infections.

## Introduction

The rise of antimicrobial resistance has become one of the greatest threats to human health (De Oliveira et al., 2020), compromising the ability to treat and prevent infections effectively. In 2019 alone, antibiotic-resistant bacterial infections were directly responsible for 1.27 million deaths, with around 5 million deaths associated with drug-resistant infections worldwide (Murray et al., 2022). This figure likely underestimates the true burden, given underreporting in some parts of the world. In the United States, there are over 2.8 million antimicrobial-resistant infections annually. Antimicrobial resistance is associated with substantial healthcare costs, totaling more than $4.6 billion each year (Nelson et al., 2021). There is significant evolutionary pressure driving the emergence of antimicrobial resistance due to the widespread use of antibiotics in human healthcare, veterinary medicine, and agricultural practices (Blair et al., 2015). There are resistance mechanisms for every antibiotic in use today and very few new drugs being actively developed (Blair et al., 2015). Even for newly developed antibiotics, resistance has rapidly emerged in clinical settings (Castanheira et al., 2020; Dadashi et al., 2021; Van Asten et al., 2021; Karakonstantis et al., 2022). By 2050, it is estimated that antimicrobial resistance will be responsible for 39 million deaths annually, with a total of 169 million deaths attributable globally (Naghavi et al., 2024). There is an urgent need for new non-antibiotic-based treatment strategies as developing new antibiotics is a challenging, slow, and costly process combined with bacteria’s ability to rapidly develop resistance to both new and existing drugs (**Table 1**).

**Table 1:** Key points.

Antimicrobial resistance (AMR) is a major global health threat, already causing millions of deaths annually and projected to cause up to 39 million deaths per year by 2050.
The gut is a major reservoir of resistant bacteria, including drug-resistant Enterobacterales (ESBL-E, CRE) and vancomycin-resistant enterococci (VRE).
Colonization with multidrug-resistant organisms in the gut can progress to invasive infections, such as bloodstream infections and urinary tract infections.
Strategies such as fecal microbiota transplantation, bacteriophage therapy, and antimicrobial peptides represent potential non-antibiotic approaches to remove drug-resistant bacteria from the gut before they cause infection.
Improving and advancing these approaches could play a key role in combating the rising threat posed by resistant bacterial infections.

The gut is a major source of drug-resistant bacteria. The gut contains a largely diverse and dense population of bacteria, with a significant portion of them harboring resistance against a broad range of antibiotics. The diverse microbial community plays a critical role in maintaining various physiological functions that are vital for overall human health (Ogunrinola et al., 2020). Among these functions is the process of colonization resistance, where gut microbes help defend against multidrug-resistant bacteria by outcompeting potential pathogens for space and nutrients or by enhancing host immune responses, thereby suppressing the colonization of harmful facultative anaerobes like Enterobacterales. Disruptions to the healthy gut microbiota, especially during antibiotic treatment, leads to the selection of drug-resistant bacteria. Several mechanisms lead to the persistence and spread of resistance genes within the intestinal microbiome. Bacteria develop resistance through mutation-driven mechanisms. These arise when spontaneous or antibiotic-induced mutations alter key bacterial targets which reduce the drug effectiveness (Irfan et al., 2022). For example, mutations can modify the antibiotic’s target site so the drug can no longer bind effectively, decrease membrane permeability to limit antibiotic entry, or increase the expression of efflux pumps that expel the drug from the bacterial cell (Smith et al., 2023). Under antibiotic selection pressure, bacteria carrying such advantageous mutations survive and multiply, while susceptible strains are eliminated (Hasan et al., 2021). Over time, these resistant traits become stable within the population, expanding the gut resistome (total collection of antibiotic resistance genes found in the bacteria within the gut microbiome), contributing to the long-term persistence and spread of antibiotic resistance. Another key process is horizontal gene transfer (HGT), which allows bacteria to exchange antibiotic-resistant genetic material (Michaelis and Grohmann, 2023). HGT can occur through 1. conjugation, where plasmids are transferred *via* direct cell-to-cell contact; 2. transformation, where bacteria uptake free DNA fragments from the environment; and 3. transduction, where bacteriophages mediate gene transfer between bacterial hosts (Munita and Arias, 2016; de Sousa et al., 2023). Together, these mechanisms promote the rapid spread of resistance genes, including multidrug resistance, across diverse bacterial populations within the gut.

**Figure 1:**
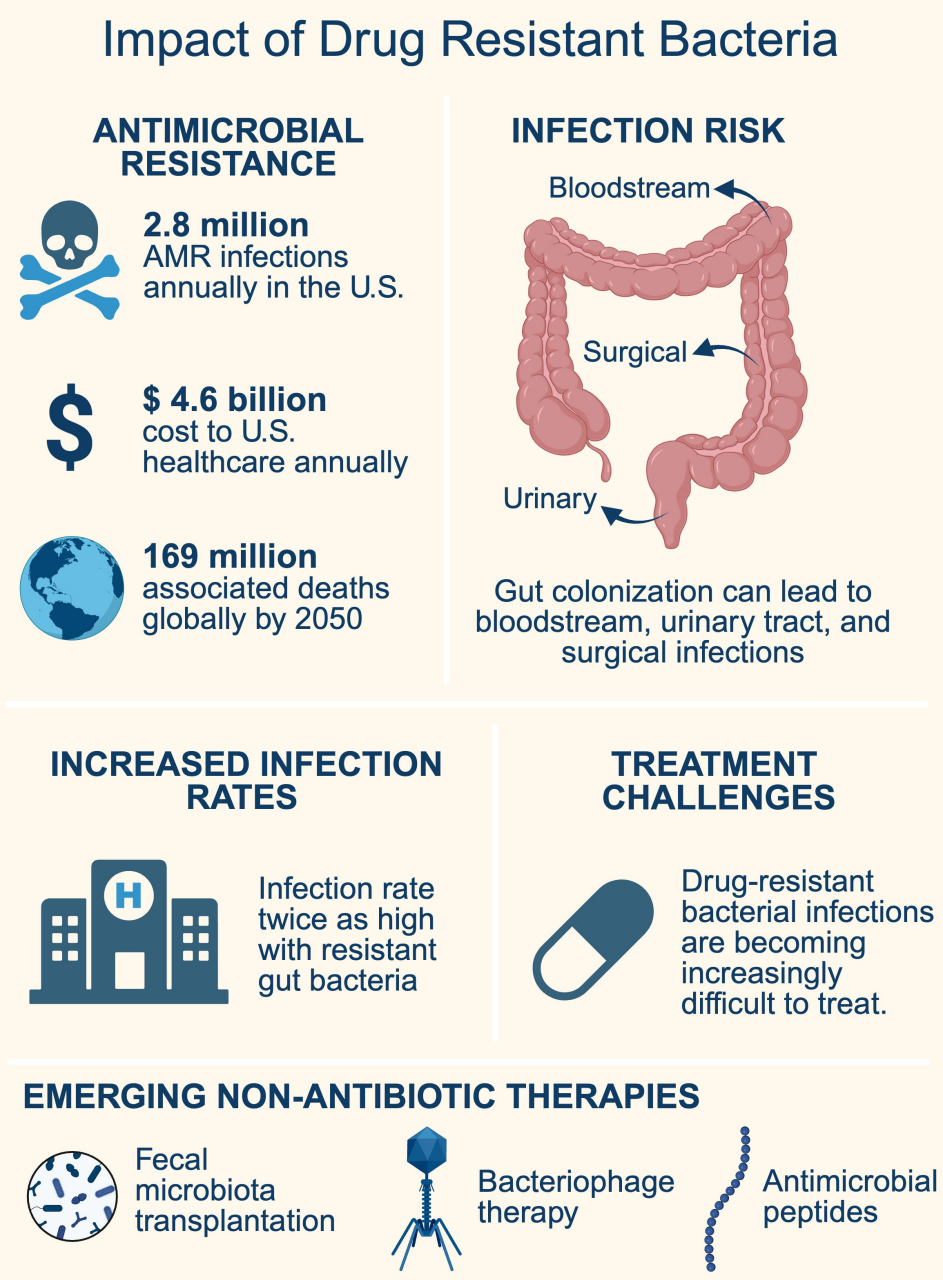
Burden of drug resistant bacterial infections.

Gut colonization with drug-resistant bacteria increases the likelihood of developing invasive infections. For example, in a retrospective study, it was observed that patients with multiple myeloma who were colonized with antibiotic-resistant bacteria (ARB) before undergoing autologous stem cell transplantation (auto-SCT) had significantly higher overall infection rates compared to those who were not colonized. Specifically, the study highlighted that 52% of colonized patients experienced infections, compared to 26% of non-colonized individuals (Bilinski et al., 2016). The study concluded that colonization of the gut by ARB within three months before auto-SCT is associated with a higher incidence of post-transplant infections, primarily bloodstream infections. It is estimated that up to 80% of gut bacteria have resistance to at least one antibiotic (Hu et al., 2014). Among antibiotic-resistant bacteria that reside in the gut, Gram-negative organisms that produce extended-spectrum β-lactamases and carbapenemases, as well as Gram-positive vancomycin-resistant enterococci, are considered major threats. Multiple studies have shown that intestinal colonization with these multidrug-resistant organisms, extended-spectrum β-lactamase producing Enterobacterales (ESBL-E), carbapenem-resistant Enterobacterales (CRE), and vancomycin-resistant enterococci (VRE), often progresses to subsequent infection. For example, a review of 17 systematic reviews and meta-analyses found that 22% of patients colonized with ESBL-E or CRE developed subsequent infection (Ling et al., 2021). In a 1-year ICU cohort of 498 patients, nearly half of *Klebsiella pneumoniae* infections were linked to prior gut colonization (Gorrie et al., 2017). In a prospective cohort of liver transplant candidates, 27% of those colonized with VRE developed an infection with VRE after transplantation (McNeil et al., 2006). This also holds true among immunocompetent populations. A recent meta-analysis reported that individuals colonized with multidrug-resistant Gram-negative bacteria (ESBL-E and CRE) had a pooled infection incidence of approximately 22%, compared to 2–5% among non-colonized controls (Woodhouse et al., 2025). Similarly, another study found that intestinal colonization with multidrug-resistant Enterobacterales was independently associated with subsequent bloodstream infection in hospitalized patients (Loukili et al., 2025). These studies highlight that colonization with resistant organisms frequently precedes an invasive infection (**Figure 1**).

Given that these multidrug-resistant organisms can leave the gut and cause difficult-to-treat infections such as bloodstream, urinary tract, and surgical site infections, new strategies are needed to decolonize the gut of these organisms and prevent their spread. This review focuses on emerging non-antibiotic approaches designed to eliminate drug-resistant bacteria from the gut before they lead to serious infections. Strategies discussed include fecal microbiota transplantation, bacteriophage therapy, antimicrobial peptides, probiotics, and dietary interventions (**Table 2**). The mechanisms underlying the development of drug resistance, approaches to prevent resistance, and the use of antibiotics for gut decolonization have been well reviewed elsewhere (Tacconelli et al., 2019; Davido et al., 2025; Dongre et al., 2025).

**Table 2:** Non-antibiotic strategies for drug resistant bacteria.

Strategy	Advantages	Limitations
Fecal microbiota transplantation	Proven effective for recurrent C. difficile. Restores gut microbial diversity and colonization resistance. Evidence of ESBL-E, CRE, and VRE decolonization in studies. May reduce infections and speed decolonization. Flexible delivery routes (colonoscopy, enema, oral capsules).	Variable and inconsistent success rates. Some patients remain colonized or re-colonized. Mechanisms are not fully understood. Risk of transmitting pathogens despite screening. Lack of standardized protocols. Regulatory and logistical challenges. Invasive methods not suitable for all patients. FMT durability is vulnerable to antibiotic exposure. Does not represent the full composition of the gut microbiome.
Bacteriophage therapy	Highly specific, target only certain bacteria, sparing commensals. Preserve microbiome balance and reduce cross-resistance. Effectiveness shown in clinical case reports (e.g., MDR Klebsiella). Can be delivered orally, rectally, or topically with minimal side effects. Growing interest as antibiotic alternatives.	Risk of horizontal gene transfer, especially with lysogenic phages. Bacterial resistance to phages can emerge (e.g., receptor changes, biofilms). Narrow host range requires strain-specific phages, slowing treatment. Limited clinical evidence with safety and efficacy not fully established. Regulatory and standardization hurdles delay widespread approval. Therapy customization is often needed, making urgent use difficult.
Antimicrobial peptides	Broad-spectrum antimicrobial activity. Naturally occurring, part of innate immunity across animals, plants, and microbes. Multiple mechanisms of action, especially membrane targeting, reduce resistance risk. Bacteriocins show promise in gut models, clearing VRE without major microbiome disruption.	Susceptible to degradation in the GI tract (enzymes, pH). Short half-life and low oral bioavailability limit effectiveness. Potential non-specific activity may disrupt gut microbiome balance. Risk of immunogenicity or cytotoxicity in host tissues. Thousands of AMPs exist, but few are well-characterized for clinical use.
Probiotics	Support a healthy gut microbiome balance. Generally safe and well-tolerated. Probiotics may reduce persistence of resistant bacteria. Certain strains show higher decolonization potential. Prebiotics nourish beneficial bacteria and may enhance probiotic effects.	Human studies on MDRO decolonization are limited. Outcomes vary widely depending on strain and pathogen. Efficacy may be overestimated. Benefits are generally modest compared to other strategies. Long-term effects and optimal combinations remain unclear.
Dietary Interventions	Diet can shape the gut microbiome and resistome. High-fiber intake linked to fewer antibiotic resistance genes (ARGs). Diets rich in whole grains, prebiotics, and traditional foods may reduce resistance. Lactose-free diet shown to lower VRE colonization in transplant patients. Non-invasive and broadly applicable intervention.	Effects are modest compared to other strategies. Benefits vary widely. Human studies are limited. Unlikely to fully clear MDROs on its own. Best used as an adjunct to other therapies.

## Fecal microbiota transplantation

Fecal microbiota transplantation (FMT), known for its effectiveness against recurrent *Clostridium difficile* infections (Johnson et al., 2021; Kelly et al., 2021; Shirley et al., 2023), is gaining attention as a potential method for decolonizing intestinal multidrug-resistant bacteria (Nhu and Young, 2023). FMT is a procedure that involves the infusion of processed stool from a healthy donor to the gastrointestinal tract of a recipient. Theoretically, FMT works by restoring a healthy and diverse microbiome, which enhances colonization resistance against pathogens (resistome). FMT involves transferring stool from a carefully screened healthy donor into the gastrointestinal tract of a recipient with microbial dysbiosis, with the goal of restoring a balanced gut ecosystem. The underlying principle is restoring the gut ecosystem by reintroducing diverse and functionally competent microbial communities that can outcompete pathogens and re-establish colonization resistance. The strongest evidence of efficacy is seen in recurrent *Clostridioides difficile* infection, where FMT achieves cure rates of 80% (Gupta et al., 2016; Xiao et al., 2020; Kelly et al., 2021).

Successful outcomes depend not only on the recipient’s microbial environment but also on the characteristics of the donor microbiota. Clinically, FMT relies on healthy adult donors, typically between 18–50 years of age. These individuals undergo extensive medical screening and laboratory testing to exclude infectious agents, gastrointestinal disorders, metabolic diseases, and recent antibiotic exposure (Gupta et al., 2016; Mehta et al., 2022). Although donor age and health status significantly vary between stool banks, younger adults with high microbial richness and low inflammatory risk profiles are often preferred. Some data suggest that higher microbial diversity in donor stool correlates with improved FMT engraftment and therapeutic outcomes (Porcari et al., 2023). The use of infant or pediatric donors is uncommon and largely experimental. Some pediatric studies have raised concerns that adult donor microbiota could alter the developmental trajectory of the child’s gut ecosystem. Nonetheless, limited animal and pediatric evidence suggest age-matching between donor and recipient may improve compatibility (MacLellan et al., 2021).

Widely available preclinical evidence from animal models provides mechanistic insight into how FMT restores colonization resistance against multidrug-resistant organisms. In antibiotic-treated mice colonized with VRE, donor microbiota containing Barnesiella spp. cleared intestinal VRE (Ubeda et al., 2013; Caballero et al., 2017). Similar studies involving carbapenem-resistant Klebsiella pneumoniae (CRKP) demonstrated that recolonization with a healthy microbiota through FMT significantly reduced the pathogen burden (Osbelt et al., 2021; Yang et al., 2025). These studies additionally help explain one of the major limitations of FMT, particularly, the variability observed in human trials. Specifically, engraftment success depends strongly on frequency of administration, antibiotic conditioning, and donor compatibility (Staley et al., 2017; Le Roy et al., 2019; Bokoliya et al., 2021). Mouse models also reveal that FMT can transfer undesirable traits, including antimicrobial-resistance genes (Liu and Wang, 2020; Yadegar et al., 2023). Collectively, these preclinical findings have provided the foundation for subsequent clinical investigations evaluating FMT for decolonization of multidrug-resistant organisms in humans.

Several small case reports, case series, and cohort studies have evaluated FMT for decolonization of multidrug-resistant organisms (MDROs), with reported success rates varying widely. For ESBL-E, Bier et al. described successful decolonization of *K. pneumoniae* in a patient with recurrent urinary tract infections. During the 18 months following FMT, the patient did not experience new infections with ESBL-producing organisms (Bier et al., 2023). In another study of 15 patients colonized with ESBL-E, FMT achieved clearance in 3 patients after the first transplant and 6 patients after a second transplant, with response associated with number of procedures (Singh et al., 2018). For CRE, Silva et al. reported decolonization in 10 of 13 carriers, most of whom also had refractory C. difficile infection. It should be noted that 8 of 21 CRE carriers were excluded in this study due to incomplete post-FMT testing (Silva et al., 2020). Bar-Yoseph et al. achieved CRE clearance in 9 of 15 using oral capsules, Lee et al. observed decolonization in 5 of 10 patients at 3–5 months, and Liu et al. documented clearance in 3 patients with longitudinal microbiome sequencing (Bar-Yoseph et al., 2021; Lee et al., 2021; Liu et al., 2022). Saidani et al. retrospective case-control suggested high CRE clearance (8/10 vs. 2/20) but authors suggest the high success rate was due to a pretreatment regimen consisting of chlorhexidine, bowel lavage, and non-absorbable antibiotics (Huttner et al., 2019). A multicenter study of 20 patients with CRE, FMT achieved clearance in 4 at two weeks and 8 at three months (Davido et al., 2024). In another multicenter study that included both CRE and VRE carriers found decolonization in 4 of 8 CRE and 7 of 8 VRE carriers after three months of receiving FMT (Dinh et al., 2018). In a study of VRE carriers, three patients remained colonized at one month, and by three months FMT achieved decolonization in 7 of 8 patients (Davido et al., 2019). In a cohort of 35 patients with CRE, VRE, or both, FMT achieved decolonization in 24 within one year of treatment (Seong et al., 2020).

More rigorous trials have shown inconsistent outcomes. In a randomized controlled trial of 11 renal transplant recipients, Woodworth et al. compared bowel preparation plus FMT with bowel preparation alone for MDRO eradication. FMT led to faster decolonization and reduced recurrent urinary tract infections (Woodworth et al., 2023). On the other hand, a multicenter randomized clinical trial found no significant difference between FMT and controls in ESBL-E/CRE carriers (41% vs. 29%) (Huttner et al., 2019). In a nonrandomized pilot trial of 42 long-term acute care hospital patients with intestinal MDRO colonization, none of the 10 FMT recipients developed bloodstream infections within six months, compared with 19% of 32 controls, though the difference was not statistically significant (Woodworth et al., 2025).

While benefits of FMT have been documented, a significant subset of patients fail to achieve decolonization and/or prevention of infection, contributing to variability in clinical outcomes. Moreover, the mechanisms underlying its protective effects in MDRO gut colonization remain poorly understood. FMT is associated with several challenges, including safety risks, lack of standardization, inconsistent long-term effectiveness, and the potential transmission of infectious agents due to variability in donor stool composition, despite rigorous screening protocols. The invasiveness of certain delivery methods (e.g., colonoscopy) and regulatory issues surrounding standardization further complicate its widespread application. Finally, although FMT can transiently reduce MDR colonization, concerns remain about the long-term stability of the transplanted microbiota and the possibility of re-colonization with resistant organisms, highlighting the need for further investigation (Karimi et al., 2024; Yadegar et al., 2024).

Although the incidence of serious adverse effects may be underreported, FMT poses minimal risk in immunocompetent patients, with only rare reports of serious adverse events such as hospitalization, pathogen transmission, or death. Systematic reviews have found that while the overall incidence of severe adverse events from FMT remains low (around 1%), serious infections are more frequently observed in immunocompromised patients, particularly those with underlying malignancies, solid organ transplants, or chemotherapy-induced immunosuppression (Wang et al., 2016). Immunocompromised patients face an increased risk of adverse outcomes from the transfer of live microorganisms (Alrabaa et al., 2017; Lin et al., 2018). That said, the data on the effects of FMT remain limited given that most trials exclude this high risk population (Karimi et al., 2024).

The composition and diversity of the gut microbiome vary significantly along the different regions of the GI tract. Spatial profiling along the gastrointestinal tract reveals substantial variation in microbial diversity, abundance, and metabolic potential between different regions (Rehman et al., 2016). Studies have demonstrated that the fecal microbiome does not fully represent the composition or functional complexity of the gut microbiome. Fecal samples mainly represent only the terminal contents of the large intestine and fail to capture the full diversity and function of the gut ecosystem (Ahn et al., 2023; Ahn et al., 2025). Comparative analyses reveal that fecal microbiota exhibit lower microbial richness, weaker ecological networks, and reduced metabolic complexity, reflecting only transient or shed microbial populations rather than stable mucosa-associated communities (Ahn et al., 2023). Because fecal material represents only a subset of the gut microbiome, mainly distal luminal bacteria, FMT may not fully restore the region-specific, mucosa-associated microbial communities required for durable colonization resistance. This difference might explain the inconsistent decolonization of drug-resistant bacteria with FMT.

## Bacteriophage therapy

Bacteriophages, commonly known as phages, are viruses that infect and kill bacteria. Phages are specific to bacteria and do not infect or directly damage human cells. Phages were independently discovered around 1915–1917 by Frederick Twort (1915) and Félix d’Hérelle (1917) and were recognized as possible antibacterial agents. However, the rise of antibiotics in the 1940s led to a decline in phage research, but the growing issue of antibiotic resistance sparked renewed interest in phages as a potential alternative to combat resistant bacteria (Sulakvelidze et al., 2001; Chanishvili, 2012; Gordillo Altamirano and Barr, 2019; Pirnay et al., 2024). Phages have two distinct life cycles: the lytic and lysogenic cycles. In the lytic cycle, the bacteriophage attaches to receptors on the host bacterial cell membrane and injects its genetic material into the bacterial cell. Ultimately, the phage hijacks the host’s cellular machinery to replicate its own genetic material and causes the host cell to lyse, thereby allowing new phages to release and infect other bacterial cells. In the lysogenic cycle, the phage DNA is incorporated into the bacterial genome instead of immediately destroying the host (Guttman et al., 2005; Ofir and Sorek, 2018).

Phages are being explored as a strategy to decolonize the gut of multidrug-resistant organisms (MDROs) and reduce the risk of subsequent invasive infections. Bacteriophage-mediated decolonization has been successfully observed both *in vivo* and in clinical settings. Most evidence supporting bacteriophage therapy against MDROs comes from mouse models. In mice colonized with CRKP, oral and intrarectal administration of lytic phage cocktails significantly reduced intestinal bacterial load (Franklin et al., 2025). A similar study demonstrated that targeted phages achieved rapid decolonization in antibiotic-pretreated mice infected with ESBL-E (Galtier et al., 2016). These preclinical findings support the translational potential of bacteriophage therapy for gut decolonization. For ESBL-producing and carbapenem-resistant *E. coli*, Javaudin et al. demonstrated that bacteriophages transiently suppress resistant *E. coli*; however, long-term efficacy of phage therapy in mice was not achieved (Javaudin et al., 2021). Mouse studies reveal additional biological and methodological limitations of bacteriophage therapy. Phage resistance can rapidly emerge through bacterial receptor modification, capsule thickening, or CRISPR-Cas9 defense mechanisms, which can lead to incomplete clearance (Touchon et al., 2016). Additionally, mouse studies have shown that lysogenic phages may facilitate horizontal gene transfer of antibiotic-resistance elements, raising serious safety concerns (de Sousa et al., 2023). Collectively, these findings underscore both the promise and the challenges of translating phage-based decolonization strategies from animal models to human applications. Clinical data on the use of bacteriophages for decolonization remain limited. Most human phage therapy reports relate to infections (e.g., respiratory, skin, urinary) rather than gut carriage/decolonization (Nir-Paz and Kuijper, 2023; Pirnay et al., 2024; Kim et al., 2025). Human studies on bacteriophages for gut decolonization are limited, with the Corbellino et al. report being among the few available. In 2020, Corbellino et al. described a case where a patient with a complex medical history, including Crohn’s disease and a multi-drug resistant (MDR) strain of carbapenemase-producing KPC-3 *Klebsiella pneumoniae*, underwent successful decolonization using a cocktail of lytic bacteriophages (Corbellino et al., 2020). The patient had recurrent urinary tract infections and sepsis, and the MDR strain persisted despite treatment with Ceftazidime-Avibactam. Phage therapy was administered orally and rectally for three weeks and showed no adverse effects and effectively eliminated the MDR strain. This study supports the potential for precise bacterial decolonization using bacteriophages. Though there is currently no phage therapy approved by the FDA in the United States, several bacteriophages are being used clinically for other types of infections plus more undergoing clinical trials.

Bacteriophages offer several promising advantages to combat antimicrobial resistance. One of the main advantages of phage therapy is its highly targeted host specificity. Phages have narrow specificity which allows for targeted treatments without harming the beneficial commensal bacteria in the body. This targeted approach helps preserve the balance of the microbiome and reduces the likelihood of cross-resistance, a common issue with broad-spectrum antibiotics. Some studies support that phages are generally well-tolerated by human hosts. Unlike broad-spectrum antibiotics, bacteriophages typically infect only a single bacterial species or even a subset of strains within a species, enabling selective elimination of pathogens while sparing the commensal microbiota (Cui et al., 2024; Sahoo and Meshram, 2024). This specificity offers multiple advantages, including reduced disruption of beneficial microbial communities, lower risk of dysbiosis, and the potential for precision therapeutics (Ross et al., 2016; Jia et al., 2023). Additionally, host-specific phages can be combined into cocktails or engineered to broaden their host range where needed, allowing for added precision and flexibility. That said, the very narrow host range also introduces practical challenges, including bacterial resistance to phages and immune neutralization of phages (Nick et al., 2022; Sawa et al., 2024). However, phage therapy faces several important limitations. A major concern is the potential for horizontal gene transfer, as lysogenic phages can spread antibiotic resistance genes, especially under the influence of phage inducers such as fluoroquinolones. Another challenge is phage resistance, since bacteria employ mechanisms like receptor alterations, extracellular polymeric substances (e.g., in *Pseudomonas*), and glycoconjugates (e.g., in Enterobacteriaceae) to block phage attachment and infection. Additional barriers include the scarcity of clinical evidence, with limited data on safety, efficacy, and long-term outcomes, hindering regulatory approval and standardization. Effective treatment often requires identifying or developing phages specific to the patient’s bacterial strain. This customization can delay therapy, making phage treatment less practical for urgent infections. These issues can sometimes be partially mitigated by the use of existing phage libraries and pre-formulated cocktails. The narrow host range of phages, complications from lysogeny, and the unpredictability of combining phages with antibiotics further restrict their therapeutic application (Uyttebroek et al., 2022; Oromí-Bosch et al., 2023; Ranveer et al., 2024). Despite these challenges, ongoing research is critical to clarify bacteriophage–bacteria interactions and realize phage therapy’s potential as a treatment option.

## Antimicrobial peptides

Antimicrobial peptides (AMPs) offer promising alternative therapeutic options to combating drug-resistant microbes due to their broad therapeutic potential. AMPs are small, catatonic, naturally occurring oligopeptides that are extensively used in medicine, food, and agriculture. They are derived from animals, plants, and microorganisms. AMPs have broad-spectrum antimicrobial activity and play a significant role in the host’s innate immune response (Mba and Nweze, 2022). Antimicrobial peptides have gained growing attention due to their various and distinct antimicrobial mechanisms, including membrane-targeting mechanisms. There are key differences between mammalian cell membranes and bacterial membranes. When AMPs interact with microbial cell membranes, they accumulate on the surface and either diffuse or undergo conformational changes, ultimately leading to rapid bacterial death (Zheng et al., 2025).

AMPs are currently being evaluated as antimicrobial agents in clinical trials (Dijksteel et al., 2021). In addition, they are also being explored for their potential role in decolonizing the gut of MDROs. Although investigations in humans in this area are scarce, several studies provide compelling preclinical evidence. Bacteriocins, a class of antimicrobial peptides and proteins produced by bacteria, have been studied for their efficacy and suitability as alternatives to traditional antibiotics. In an experimental mouse gut model, colonization with bacteria expressing bacteriocin 21 resulted in clearance of intestinal colonization by VRE, without causing significant disturbance of gut microbes (Kommineni et al., 2015). This study supports the concept that the presence of AMPs like bacteriocins in the gut may be effective in eliminating intestinal colonization by multidrug-resistant bacteria, providing a foundation to pursue clinical application of AMPs in MDRO gut decolonization.

AMPs have some limitations that would need to be addressed for successful clinical application in MDRO gut decolonization. One major concern is their susceptibility to the harsh gastrointestinal environment, including enzymatic degradation and variable pH levels, which can significantly diminish their bioavailability and efficacy upon oral administration. However, this might be addressed by modifying the peptide sequences or utilizing gut specific delivery systems. There are thousands of AMPs that have been discovered since 1939 (Kang et al., 2017).Further research is needed to identify the most suitable candidates, as issues such as non-specific antimicrobial activity that could disrupt the complex balance of the gut microbiome, immunogenicity, cytotoxicity, and short half-lives remain significant barriers that must be addressed to realize the full clinical potential of AMPs (Chen and Lu, 2020).

## Probiotics and dietary interventions

Probiotics are live microorganisms, usually bacteria or yeasts, that when consumed in adequate amounts provide benefits as they help to maintain a healthy balance in the gut microbiome. Prebiotics are non-digestible dietary components, mostly fiber, that serve as nutrients that promotes growth and function of beneficial gut bacteria (Sanders et al., 2019; Cunningham et al., 2021). Human studies showing significant benefit of probiotics and prebiotics in clearing MDROs from the gut remain limited. A recent systematic review and meta-analysis of 29 randomized controlled trials (n=2,871) evaluated the role of probiotics in decolonizing antimicrobial-resistant bacteria from the gut. Probiotic treatment was associated with reduced persistence of colonization (22% vs. 30.8% with placebo), suggesting a moderate benefit (Rahman et al., 2024). Notably, outcomes varied by both probiotic type and pathogen. For example, certain strains, such as Lactobacillus spp. and *Saccharomyces boulardii*, showed the highest decolonization rates. That said, the study was limited by the pooling of pathogens, that included C. difficile, as this might have overestimated the overall efficacy, particularly since organisms like C. difficile are more responsive to these interventions (Rahman et al., 2024).

Diet can modify the composition of the microbiome; hence, dietary strategies are being explored as potential tools to mitigate resistance. In a study involving 290 healthy participants, Oliver et al. examined how dietary patterns influence the gut microbiota and the presence of antibiotic resistance genes (ARGs) (Oliver et al., 2022). They discovered that individuals with higher fiber intake tended to have fewer ARGs in their gut microbiome. These individuals showed a greater presence of obligate anaerobic bacteria, such as those from the Clostridiaceae family, whereas those with elevated ARG levels had more Streptococcaceae and Enterobacteriaceae, which are often associated with antibiotic resistance. Interestingly, the group with lower ARG levels also consumed less protein from animal sources, particularly red meats like beef and pork. The research by Wu and colleagues revealed the potential of a gut microbiota-focused dietary strategy, rich in whole grains, prebiotics, and traditional Chinese medicinal foods, to reduce antibiotic resistance in obese children (Wu et al., 2016). A lactose-free diet has showed potential in reducing Enterococcus overgrowth and improving clinical outcomes in patients undergoing allogeneic hematopoietic stem cell transplantation (allo-HCT) and other cellular therapies. Lactose removal from the diet decreased VRE colonization from 16% prior to intervention to 3.6% in those on a lactose-free diet after 3 months (Morello et al., 2024). Diet appears to have some benefits, but it is unlikely to be sufficient on its own for decolonizing drug-resistant organisms, but rather in combination alongside other interventions.

## Conclusion

Antimicrobial resistance is a growing and serious public health threat that urgently requires innovative therapeutic strategies to combat multidrug-resistant pathogens. The gastrointestinal tract, as a major reservoir for resistant bacteria, represents a promising target for interventions aimed at preventing colonization and subsequent infection. Emerging approaches, including FMT, bacteriophage therapy, dietary interventions, and antimicrobial peptides, offer promising avenues, yet each faces important challenges such as clinical validation, optimization of delivery, and regulatory hurdles. While FMT has shown clinical promise, the mechanism by which it exerts its protective effects remains unclear, and studies to date have reported varying results. Critical questions remain (**Table 3**), including whether antimicrobial peptides can be effectively targeted to the gut, whether phages can consistently and selectively decolonize multidrug-resistant organisms without disrupting the microbiota, how individual microbiome variability influences phage efficacy, and how FMT preparations can be standardized across sites. Addressing these challenges through continued research will be essential to refine these novel strategies and translate them into effective and safe clinical tools to combat antimicrobial resistance.

**Table 3:** Outstanding questions.

Can antimicrobial peptides be delivered effectively and safely to the gut?
How does microbiome variability affect phage efficacy in decolonizing MDROs?
What are the mechanisms by which FMT exerts protective effects in MDRO decolonization?
How can FMT preparations be standardized across studies and clinical centers?
What is the long-term stability of these interventions, and do they prevent re-colonization?
